# Effects of an individualized analgesia protocol on the need for medical interventions after adenotonsillectomy in children: a randomized controlled trial

**DOI:** 10.1186/s12871-021-01263-3

**Published:** 2021-02-08

**Authors:** Jian Guo, Peijun Zhuang, Kun Liu, Yuanyuan Wan, Xuan Wang

**Affiliations:** Department of Anesthesia, Children’s Hospital of Fudan University, 399 Wanyuan Road, Shanghai, 201102 China

**Keywords:** Adenotonsillectomy, Children, Individualized protocol, Medical interventions

## Abstract

**Background:**

It has been proposed that the dose of rescue opioids should be individually titrated to the severity of obstructive sleep apnea after adenotonsillectomy. However, a sleep study is not always available before adenotonsillectomy. This randomized, controlled and blinded trial evaluated a strategy of pain control individualized to the results of a fentanyl test, rather than the results of polysomnography, in children after adenotonsillectomy.

**Methods:**

A total of 280 children (3–10 years old) undergoing elective adenotonsillectomy were randomized into an individualized protocol (IP) group or a conservative protocol (CP) group. All patients received a fentanyl test before extubation. Pain was assessed every 10 min in the recovery room, and rescue morphine was given when the Children’s Hospital of Eastern Ontario Pain Scale (CHEOPS) score was > 6. In the IP group, the dose of rescue morphine was individualized to the results of the fentanyl test (10 μg/kg in the case of a positive result and 50 μg/kg in the case of a negative result). In the CP group, the dose was fixed (25 μg/kg). The primary outcome was the percentage of patients requiring at least one medical intervention. The secondary outcome was the median duration of CHEOPS scores > 6.

**Results:**

Fewer patients in the IP group than in the CP group required medical interventions [11.9% (16/134) vs 22.3% (29/130), *P* = 0.025]. The median duration of CHEOPS scores > 6 was shorter in the IP group than in the CP group [20 (95% CI: 17 to 23) min vs 30 (95% CI: 28 to 32) min, *P* <  0.001].

**Conclusions:**

Compared with a conservative dosing approach, this individualized protocol may improve analgesia without a significant increase in respiratory adverse events.

**Trial registration:**

ClinicalTrials.gov NCT02990910, registered on 13/12/2016.

## Background

In recent years, many children have presented for elective adenotonsillectomy (T&A) surgery with a tentative diagnosis of obstructive sleep apnea (OSA) [1]. It has been reported that recurrent hypoxemia in children is associated with increased analgesic sensitivity to opioids [2]. Further research found that more medical interventions were required after T&A only in children with severe OSA, and no increased risk was found in nonsevere cases [3]. In order to reduce the incidence of respiratory complications following T&A, a management strategy of pain control individualized to the severity of OSA was also recommended in the same report.

However, the dose of rescue opioids cannot be titrated in an individualized manner to the severity of OSA after T&A unless polysomnography (PSG) assessment has been performed before surgery. Unfortunately, sleep studies are not always accessible to all children scheduled for elective T&A [4]. In such cases, it seems prudent to assume that a child is more sensitive to opioids than usual and to use opioids judiciously when the child presents for T&A with pharyngeal obstruction. The conservative strategy, which is a cautious dosing approach to the titration of rescue opioids, can attenuate postoperative respiratory complications but may delay the control of pain.

It has been noticed that a small dose of fentanyl depresses spontaneous ventilation more profoundly in children with OSA than in healthy children [5]. Lerman has proposed that spontaneous ventilation can be used during anesthesia for T&A, and the capnogram can be monitored as a marker of opioid sensitivity when small incremental doses of opioids are given for titration [6]. Previously, we found that children whose respiratory rate decreased more than 50% after a fentanyl test required reduced amounts of morphine for similar pain scores after T&A [7].

This information suggests that it would be feasible to base an individualized protocol on the results of the fentanyl test. To determine whether this individualized protocol is helpful in the absence of a PSG study, we hypothesized that this individualized protocol, compared with a conservative protocol, could reduce the need for medical interventions among children in the recovery room after T&A and that the control of pain could also be improved.

## Methods

### Participants

This single-center, randomized, controlled study was approved by the Ethics Committee of the Children’s Hospital of Fudan University (Ref: 2016189) and was registered on ClinicalTrials.gov (NCT02990910, registered on 13/12/2016). The study was performed according to the Declaration of Helsinki Criteria, and this manuscript adheres to the CONSORT guidelines. Children 3–10 years old with American Society of Anesthesiologists Classification (ASA) I–II undergoing elective T&A based on a parental report of snoring and an otolaryngology evaluation that confirmed pharyngeal obstruction (tonsillar hypertrophy score ≥ 1 based on a standardized scale of 0–4) were eligible for this study. Children with recent upper airway infection, cognitive dysfunction, asthma, obesity (BMI > 95th percentile for age), or craniofacial malformations were excluded. The patients’ parents or guardians received written information about the study protocol, had ample time to decide on their children’s participation, and signed the written informed consent form before the patients were included in the study. The included patients were allocated to two groups, namely, the individualized protocol (IP) and conservative protocol (CP) groups.

### Randomization

Envelopes containing grouping information were prepared in advance by someone who had no further involvement. The grouping information was determined by a computer-generated simple (nonblock) random number sequence with a 1:1 allocation ratio (SPSS 23.0). The third author (K.L.) opened the envelopes in order of enrollment and placed the children into the corresponding groups. She was the only one with access to the randomization information. All the study medications were prepared by her. The anesthesiologist (J.G.) who performed the fentanyl test was blinded to the randomized allocations. The anesthesiologist (P.Z.) who took care of the patients in the recovery room and assessed the outcomes was blinded to both the group allocation and the results of the fentanyl test. The randomization was broken only following completion of subject enrollment and all data collection.

### Anesthesia protocol

All children underwent routine fasting before surgery and received no premedication. Intravenous access was secured prior to arrival in the operating room. Regular monitoring was performed in the operating room. After preinduction assessment, anesthesia was induced with 50% nitrous oxide (N_2_O) in oxygen, and sevoflurane was added 1 min later. The inspired concentration of sevoflurane was gradually increased to 8%. After loss of the eyelash reflex, N_2_O was discontinued, and the inspired concentration of sevoflurane was reduced to 5%. Children who experienced airway obstruction were given continuous positive airway pressure (CPAP) at 5–10 cmH_2_O, and manual ventilation was applied if apnea occurred. Two minutes later, propofol 2 mg/kg was given intravenously to facilitate tracheal intubation. The lungs were ventilated by pressure-controlled ventilation after intubation, and the end-tidal carbon dioxide concentration (EtCO_2_) of the patient was kept within 35–45 mmHg. Anesthesia was maintained with sevoflurane and N_2_O in oxygen at the discretion of the attending anesthetist. In addition, 30 mg/kg rectal acetaminophen (maximum 600 mg) as well as intravenous dexamethasone 0.25 mg/kg (maximum 10 mg) was administered before surgery. During the last 15 min of the procedure, the respiratory rate of the ventilator was reduced to slightly increase the EtCO_2_ concentration, and the ventilator was turned off if the patient began to breathe spontaneously.

### Fentanyl test

After removal of the Boyle–Davis mouth gag, both sevoflurane and N_2_O were discontinued. When the end-tidal sevoflurane concentration decreased to 0.8%, as long as the child was breathing regularly, the fentanyl test was performed by the first author (J.G.). The patient’s initial respiratory rate was recorded as the pretest frequency, followed by intravenous injection of 1 μg/kg fentanyl. The investigator recorded the lowest respiratory rate of the patient within the next 3 min as the posttest frequency. Patients with a posttest frequency more than 50% below the pretest frequency were considered positive, and patients with a posttest frequency greater than or equal to 50% were considered negative. If the child was aroused accidentally by stimuli before the test was finished, he or she was withdrawn from the study. The result of the fentanyl test for each patient was delivered to the third author (K.L.), who prepared the rescue morphine for each patient according to the result of the fentanyl test.

After the fentanyl test, the “no touch” technique was used for extubation, and absolutely no stimulation was given to the patient before extubation [8]. The trachea was extubated when the child was awake (spontaneous eye opening, purposeful movement, or end-tidal sevoflurane concentration <  0.3%).

Patients were sent to the recovery room after extubation. Pain was assessed immediately upon arrival in the recovery room and every 10 min thereafter using the Children’s Hospital of Eastern Ontario Pain Scale (CHEOPS) by the second author (P.Z.), who knew neither the randomization results nor the fentanyl test results. Intravenous morphine was given to the patients if they scored > 6 on the CEHOPS, and this treatment was continued until the CHEOPS score was ≤6. The morphine for each rescue dose was prepared in syringes according to the randomized grouping and fentanyl test results by the third author (K.L.), who did not participate in pain assessment. The rescue morphine was prepared in a volume of 0.1 ml/kg. The dose of morphine could not be determined from the appearance of the syringe.

### Intervention

In the IP group, patients were given 10 μg/kg morphine at a time if the result of the fentanyl test was positive and 50 μg/kg morphine at a time if it was negative. In the CP group, all children were given 25 μg/kg morphine at a time, regardless of the results of the fentanyl test.

The second author (P.Z.) not only assessed the pain of each patient and decided on the administration of rescue morphine but also recorded any medical intervention the patients received in the recovery room. Each patient was monitored in the recovery room for approximately 60 min, and upon achieving a CHEOPS score ≤ 6 and a modified Aldrete score ≥ 9, the patient was sent back to the regular ward.

### Primary and secondary outcomes

The primary outcome was the percentage of patients requiring at least one medical intervention for respiratory events. The medical interventions included instrumentation of the airway, bag/mask ventilation, and/or drug administration (succinylcholine, albuterol, or naloxone), which were classified as major medical interventions. The medical interventions also included repositioning of the child’s airway, chin lift/jaw thrust maneuvers, and/or escalation to an oxygen mask, which were classified as minor medical interventions. The elements used to define the interventions required for respiratory events were adopted from Raghavendran’s report [3]. Since blow-by oxygen was delivered to all children recovering from anesthesia, escalation to an oxygen mask implied that oxygen was required in a more reliable way to treat an episode of SpO_2_ < 95%.

The secondary outcome of this study was the median duration of CHEOPS scores > 6.

In order to determine whether pain relief was compromised for positive-result patients or whether the need for medical interventions for negative-result patients was increased in the IP group because of the alteration of the dose of morphine, subgroup analysis was performed for the primary outcome, the secondary outcome and cumulative morphine consumption.

### Statistical analysis

A pilot study revealed that the percentage of patients requiring respiratory medical intervention was approximately 24% among patients under the conservative protocol after T&A. The individualized protocol based on the result of the fentanyl test reduced the percentage by 50%; an estimated sample size of 126 subjects per group was required (α = 0.05, β = 0.2). Considering that some study subjects might not be able to successfully complete the research program, the sample size was increased by approximately 10% based on the formula calculation. For this reason, the study included 140 children in each group.

All data were analyzed according to an intention-to-treat principle. For the primary outcome, differences between two groups were assessed with chi-square statistics. For the secondary outcome, Kaplan–Meier survival curves were generated with equality of survivor functions between the IP and CP groups and compared with a log-rank test. A *P-*value < 0.05 was used to define statistical significance.

Subgroup analysis (of positive and negative patients) between the two arms was performed for the primary outcome, the secondary outcome and cumulative morphine consumption. Cumulative morphine consumption was assessed with a nonparametric test. Because the number of independent dimensions was 4, we applied a Bonferroni correction to adjust for multiple comparisons. Therefore, a *P-*value < 0.05/4 was used to define statistical significance for the subgroup analysis. Statistical analysis was performed with SPSS 23.0 (Statistical Package for the Social Sciences, IBM SPSS Inc., Chicago, IL, US).

## Results

**Fig. 1:**
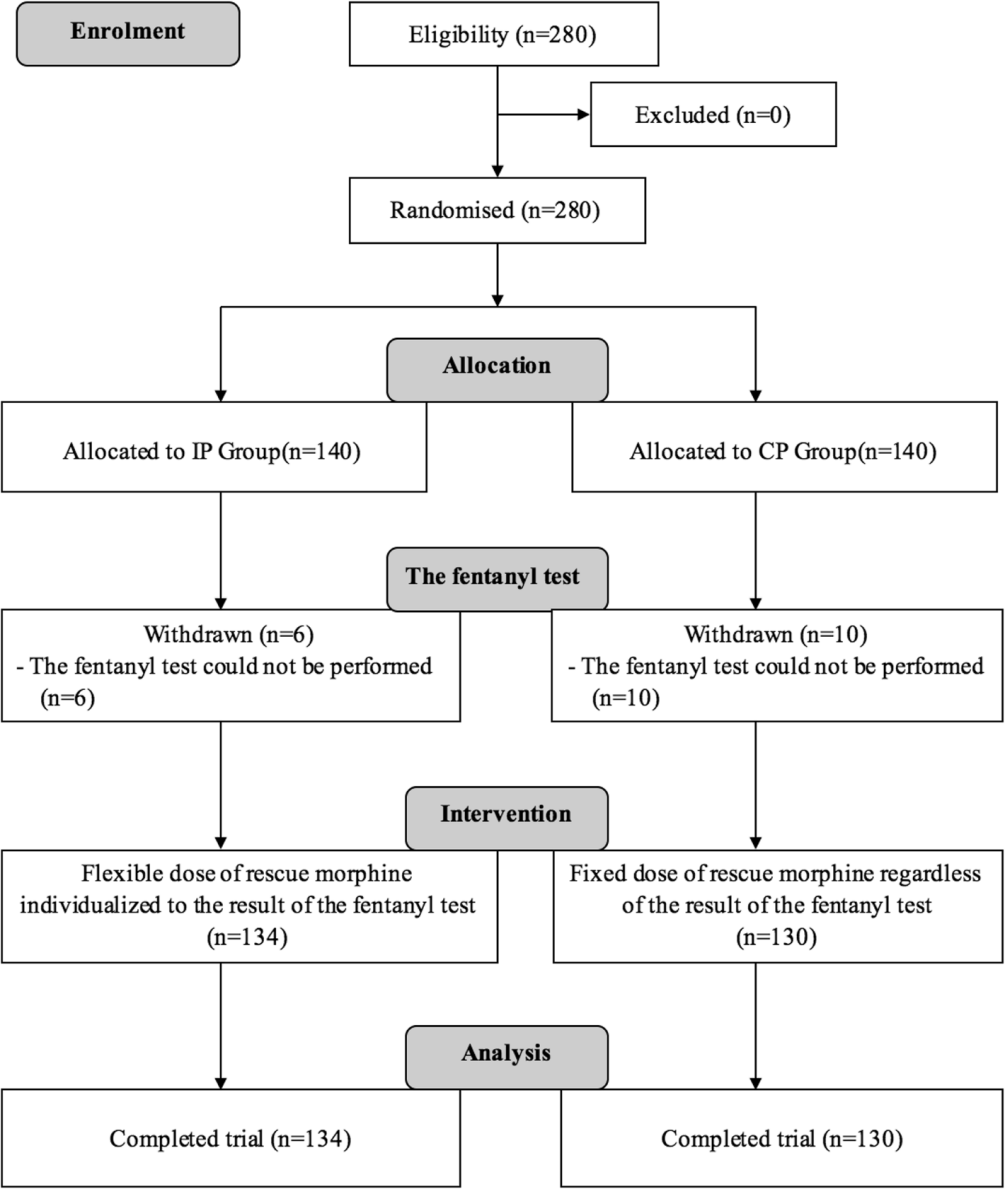
CONSORT diagram. IP, individualized protocol; CP, conservative protocol

A total of 280 children who underwent elective T&A were included in this study. All surgeries were performed with COBLATION technology. No important harm or unintended effects were found during the study period. Six patients in the IP group and 10 patients in the CP group did not finish the fentanyl test because of coughing during the removal of their mouth gags. These 16 cases were excluded from data analysis. Ultimately, 134 cases in the IP group and 130 cases in the CP group were included in the statistical analysis (Fig. 1). Table 1 shows the characteristics of the patients analyzed as well as the results of the fentanyl test.

**Table 1:** Patient characteristics and fentanyl test results

	IP	CP	*P*
	(*n* = 134)	(*n* = 130)	
Age, years [IQR]	6 [4 to 8]	5 [4 to 6]	0.124
Male, n (%)	86 (64%)	78 (60%)	0.490
BMI, kg/m^2^ [IQR]	18 [17 to 20]	18 [16 to 19]	0.422
Time to fentanyl test, min [IQR]	40 [33 to 49]	39 [31 to 44]	0.387
RR before test, bpm (SD)	22 (3)	22 (4)	0.202
EtCO_2_ before test, mmHg (SD)	53 (5)	53 (5)	0.987
EtSevo before test, % (SD)	0.79 (0.04)	0.80 (0.03)	0.223
Positive result, n (%)	44 (33%)	46 (35%)	0.726

Medians [interquartile range], number (%) and means (standard deviation) reported, as appropriate. IP, individualized protocol; CP, conservative protocol; BMI, body mass index; RR, respiratory rate; EtCO_2_, end-tidal carbon dioxide; EtSevo, end-tidal sevoflurane concentration; IQR, interquartile range; SD, standard deviation. Time to fentanyl test was defined as the time from the start of anesthesia induction to the performance of the fentanyl test

Both the primary and secondary outcomes of this study are shown in Table 2. Compared with the IP group, the relative risk (RR) of the need for medical intervention in the CP group was 1.87, and its 95% confidence interval (CI) ranged from 1.07 to 3.27.

**Table 2:** Primary and secondary outcomes

	IP	CP	*P*
	(*n* = 134)	(*n* = 130)	
Patients requiring at least one intervention, n (%)	16 (11.9%)	29 (22.3%)	0.025
Patients requiring minor interventions			
Escalation to oxygen mask, n (%)	16 (11.9%)	29 (22.3%)	0.025
Chin lift/jaw thrust maneuver, n (%)	4 (3%)	3 (2.3%)	0.740
Repositioning of the child’s airway, n (%)	6 (4.5%)	11 (8.5%)	0.182
Oropharyngeal airway insertion, n (%)	1 (0.7%)	2 (1.5%)	0.539
Patients requiring major interventions			
Reintubation/LMA insertion, n (%)	0	0	–
Bag/mask ventilation, n (%)	0	0	–
Drug administration, n (%)	0	0	–
Median duration of CHEOPS scores> 6, min [IQR]	20 [17 to 23]	30 [28 to 32]	< 0.001

Number (%) and median [interquartile range] reported as appropriate. IP, individualized protocol; CP, conservative protocol; CI, confidence interval; IQR, interquartile range; LMA, laryngeal mask airway; CHEOPS, Children’s Hospital of Eastern Ontario Pain Scale

Table 3 shows the subgroup analysis of the patients with positive and negative results. Decreasing the dose of morphine in positive-result patients did not compromise pain control. Increasing the dose of morphine in negative-result patients did not increase the need for medical interventions but shortened the median duration of CHEOPS scores> 6.

**Table 3:** Subgroup analysis

Test results	Positive			Negative		
Group allocation	IP (*n* = 44)	CP (*n* = 46)	*P*	IP (*n* = 90)	CP (*n* = 84)	*P*
Cumulative morphine consumption, μg/kg [IQR]	30 [20 to 40]	50 [50 to 75]	< 0.001	100 [100 to 150]	75 [75 to 100]	0.264
Patients requiring at least one intervention, n (%)	5 (11.4%)	17 (37.0%)	0.005	11 (12.2%)	12 (14.3%)	0.688
Median duration of CHEOPS scores> 6, min [95% CI]	20 [18 to 28]	20 [17 to 25]	0.253	20 [15 to 20]	30 [25 to 31]	< 0.001

Median [interquartile range] and number (%) reported as appropriate. IP, individualized protocol; CP, conservative protocol; CI, confidence interval; IQR, interquartile range; CHEOPS, Children’s Hospital of Eastern Ontario Pain Scale

**Fig. 2:**
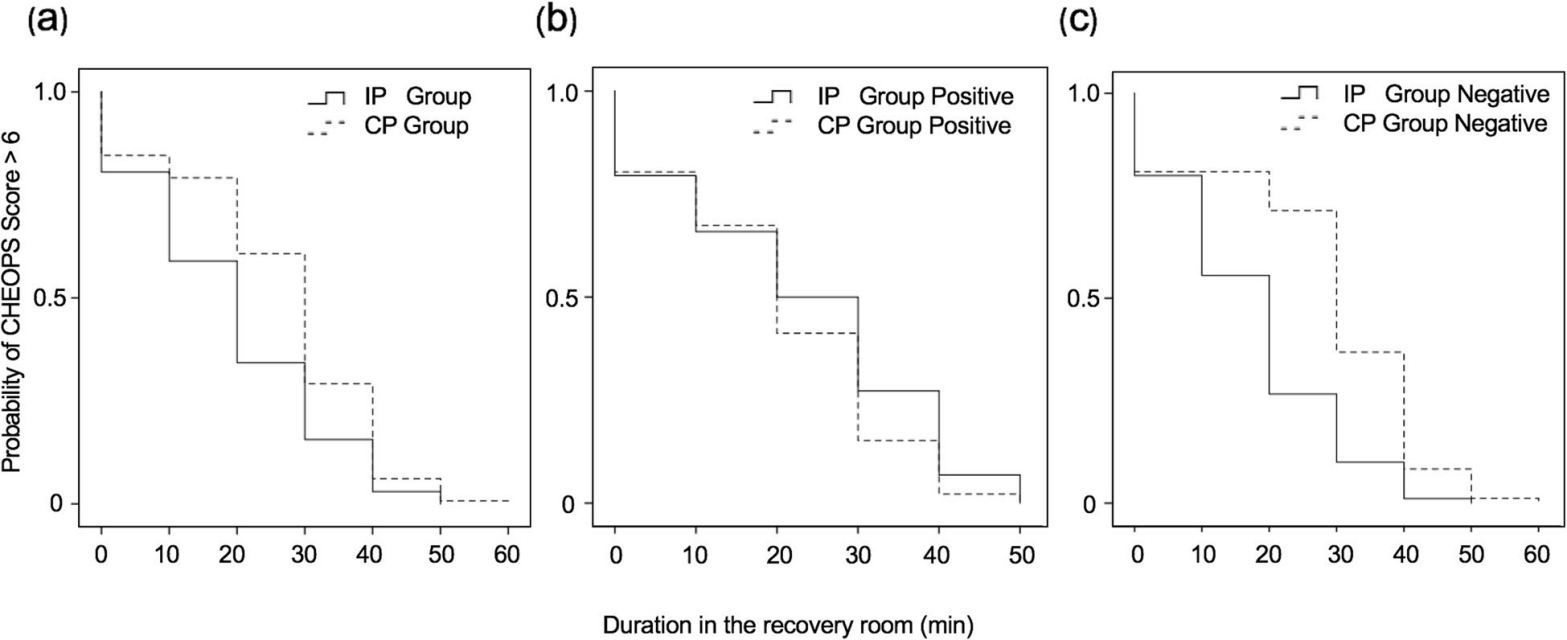
Survival curves of CHEOPS scores> 6. The probability of CHEOPS scores> 6 for all patients in the IP and CP groups, *P* < 0.001 (**a**). For the patients with positive results, *P* = 0.253 (**b**). For the patients with negative results, *P* < 0.001 (**c**). IP, individualized protocol; CP, conservative protocol; CHEOPS, Children’s Hospital of Eastern Ontario Pain Scale

Figure 2 shows the survival curve for the duration of pain (CHEOPS scores> 6) and the subgroup survival analysis for the patients with positive or negative results. Survival analysis showed that the probability of CHEOPS scores > 6 decreased more rapidly for patients in the IP group than for patients in the CP group. The difference between the two survival curves was significant (*P* = 0.000).

## Discussion

The main observation of this trial was that the percentage of patients requiring medical interventions in the recovery room was significantly lower in the IP group (11.9%) than in the CP group (22.3%). Because no major medical interventions were required in either group, this advantage should be attributed to the differential need for minor interventions between the two groups. Escalation to an oxygen mask was less frequently required in the IP group than in the CP group, which suggests that children under this individualized protocol are less vulnerable to desaturation in the recovery room after T&A. The conservative protocol uses a cautious dosing approach, prioritizing safety. Compared with this cautious protocol, the new protocol could further decrease the need for minor interventions even if no major medical interventions are required under either protocol.

The secondary outcome of this study revealed that the median duration of CHEOPS scores> 6 was shorter for patients in the IP group than for those in the CP group (20 min vs 30 min). This indicated that 50% of patients in the IP group had their pain under control 20 min after admission to the recovery room. By contrast, this was not achieved in the CP group until 30 min after admission to the recovery room. The survival curve for pain (CHEOPS > 6) declined more rapidly in the IP group than in the CP group. These results suggest that the individualized protocol allows children to achieve comfort sooner after T&A in the recovery room.

Subgroup analysis revealed that the individualized protocol, compared to the conservative protocol, provided a reduced amount of morphine to the patients with positive results and an increased amount of morphine to the children with negative results on the fentanyl test. Nevertheless, decreasing the dose of morphine in positive-result patients did not compromise pain control. Increasing the dose of morphine in negative-result patients did not increase the need for medical interventions but shortened the median duration of CHEOPS > 6. This suggests that the advantage of an individualized protocol results from the increased amount of morphine given to the low-risk individuals, identified by negative results on the fentanyl test. Further decreasing the dose of morphine given to high-risk patients may not provide additional benefits because the small dose of rescue morphine used under a conservative protocol already limits the need for major medical interventions.

Tailored dosing of analgesia according to the severity of OSA in children undergoing T&A was described previously. Isaiah et al. enrolled 65 children with PSG-proven OSA who underwent T&A. They stratified these patients into mild, moderate and severe groups based on severity, and these groups were given 10, 7, and 5 μg/kg hydromorphone, respectively, for analgesia. They found that this tailored protocol minimized perioperative respiratory adverse events in patients with severe OSA [9]. However, this conclusion was drawn without a control. Raghavendran et al. retrospectively studied adverse events during T&A in historic cohorts that presented with OSA [3]. They found that a new protocol including a reduction in opioid administration to severe cases reduced the incidence of major medical interventions by 50%. Although both studies support the judicious use of opioids in children with severe OSA as a means of attenuating respiratory events after T&A, neither of them reported whether pain control was delayed. An overnight sleep study was also required before surgery in those reports.

This study has some limitations.

This is a single-center study. Some aspects of the anesthetic protocol in this study, such as lack of premedication and late use of fentanyl, may vary from many other practices. Premedication may change the respiratory rate at the time when the fentanyl test is performed, although some data showed that it might not [10]. However, this possible effect exists not only before but also after fentanyl administration. Thus, the result of this test is relatively unlikely to be affected because it measures the relative drop in respiratory rate after fentanyl administration.

Late use of fentanyl may also be different from many other practices. Intraoperative opioid use varies, and some practitioners do not utilize any opioids in this kind of surgery, as evidenced by the survey of Roberts et al. [11]. Unconsciousness, immobility, and the control of autonomic nervous system responses to nociception can also be guaranteed with potent inhalational anesthetics only, since opioids are dispensable for general anesthesia [12]. It has been reported that late use of analgesics might provide comparable surgical conditions and superior postoperative analgesia relative to early use of analgesics in children undergoing tonsillectomy [13].

A dose of 1 μg/kg fentanyl was used in the fentanyl test for all the participants in this study. This fixed dose based solely on body weight might produce different target plasma concentrations of fentanyl in different patients because of their different developmental stages. However, according to a report by Singleton et al., the standard deviation of the measured plasma concentration of fentanyl after a fixed dose based on body weight in 1- to 9-year-old children was small and similar to that in adult patients [14]. In daily practice, dosing based on body weight is more feasible than achieving a specific target concentration. In addition, considering the convenience of the clinical situation, this study recorded the posttest respiratory rate 3 min after the administration of fentanyl. A computerized simulation (iTIVA, iOS App Store) using Ginsberg’s model [15] showed that the effect-site concentration of fentanyl was very close to its peak value in pediatric patients 3 min after a bolus of fentanyl.

The authors speculated that a 50% reduction might be an indicator of significant effects on breathing. At study inception, no published data could be used to justify the cutoff point for the fentanyl test. Ansermino et al. demonstrated that spontaneously breathing children could tolerate increasing doses of remifentanil infusion well until a 50% reduction in respiratory rate occurred [16].

Medical interventions were applied to patients during recovery at the discretion of the anesthetist who was in charge of the recovery room. This may have created an observer bias. However, the same anesthetist was assigned to take care of all the patients enrolled throughout the whole study period. This may minimize the bias.

The absence of PSG assessment before surgery in the patients recruited in this study is also a limitation. It should be noted that patients with positive results were not necessarily severe OSA patients. What the fentanyl test measured was the sensitivity to fentanyl. Nevertheless, subjects who were sensitive to fentanyl in this specific population are very likely to be severe OSA patients, according to Brown’s report [2]. There is a large volume of patients undergoing T&A in this institution. PSG is less frequently performed because of limited resources. Diagnosis is usually made according to the clinical syndrome and physical examination. However, all participants were presenting for T&A with different severities of pharyngeal obstruction; this research was driven by exactly that motivation. The purpose of this study was to evaluate a novel strategy for pain control individualized to the results of a fentanyl test when a sleep study is unavailable. It may be the sensitivity to fentanyl, rather than the real severity of OSA, that practitioners must consider when determining the dose of rescue opioids.

## Conclusion

Although all the interventions required in this study were minor, the data from this trial support the hypothesis that an individualized protocol could reduce children’s need for medical interventions in the recovery room after T&A. In conclusion, when a sleep study is unavailable before elective T&A, a management protocol for pain control can be individualized based on the results of a fentanyl test. Compared with a conservative protocol that prioritizes safety, this individualized protocol may improve analgesia for children in the recovery room after T&A without a significant increase in respiratory adverse events.

## Data Availability

Reasonable requests for access to the datasets used and/or analyzed during this study can be made to the corresponding author.

## Abbreviations

T&A: adenotonsillectomy
OSA: obstructive sleep apnea
PSG: polysomnography
IP: individualized protocol
CP: conservative protocol
N_2_O: nitrous oxide
CI: confidence interval
IQR: interquartile range
LMA: laryngeal mask airway
CHEOPS: Children’s Hospital of Eastern Ontario Pain Scale
LMA: laryngeal mask airway
CPAP: continuous positive airway pressure
EtCO_2_: end-tidal carbon dioxide concentration
